# The price of defence: toxins, visual signals and oxidative state in an aposematic butterfly

**DOI:** 10.1098/rspb.2022.2068

**Published:** 2023-01-25

**Authors:** Jonathan D. Blount, Hannah M. Rowland, Christopher Mitchell, Michael P. Speed, Graeme D. Ruxton, John A. Endler, Lincoln P. Brower

**Affiliations:** ^1^ Centre for Ecology and Conservation, College of Life and Environmental Sciences, University of Exeter, Penryn Campus, Cornwall TR10 9FE, UK; ^2^ Research Group Predators and Toxic Prey, Max Planck Institute for Chemical Ecology, Hans-Knöll-Straße 8, Jena, 07745, Germany; ^3^ Institute of Systems, Molecular and Integrative Biology, University of Liverpool, Liverpool L69 7BE, UK; ^4^ School of Biology, Sir Harold Mitchell Building, Greenside Place, St Andrews, UK; ^5^ Centre for Integrative Ecology, School of Life and Environmental Sciences, Deakin University, Waurn Ponds, Victoria 3216, Australia; ^6^ Department of Biology, Sweet Briar College, Sweet Briar, VA 24595, USA

**Keywords:** aposematism, cardenolides, resource competition, oxidative lipid damage, *Danaus plexippus*, honest signalling

## Abstract

In a variety of aposematic species, the conspicuousness of an individual's warning signal and the quantity of its chemical defence are positively correlated. This apparent honest signalling is predicted by resource competition models which assume that the production and maintenance of aposematic defences compete for access to antioxidant molecules that have dual functions as pigments and in protecting against oxidative damage. To test for such trade-offs, we raised monarch butterflies (*Danaus plexippus*) on different species of their milkweed host plants (Apocynaceae) that vary in quantities of cardenolides to test whether (i) the sequestration of cardenolides as a secondary defence is associated with costs in the form of oxidative lipid damage and reduced antioxidant defences; and (ii) lower oxidative state is associated with a reduced capacity to produce aposematic displays. In male monarchs conspicuousness was explained by an interaction between oxidative damage and sequestration: males with high levels of oxidative damage became less conspicuous with increased sequestration of cardenolides, whereas those with low oxidative damage became more conspicuous with increased levels of cardenolides. There was no significant effect of oxidative damage or concentration of sequestered cardenolides on female conspicuousness. Our results demonstrate a physiological linkage between the production of coloration and oxidative state, and differential costs of sequestration and signalling in monarch butterflies.

## 1. Introduction

Many species of animals, plants and micro-organisms possess chemical defences that reduce the likelihood of their being eaten [1]. Aposematic species, such as the monarch butterfly (*Danaus plexippus*), signal their chemical defences to predators with conspicuous warning colours [2]. In some species, the conspicuousness of the warning signal and the quantity of chemical defence are positively correlated—for example, in some dendrobatid frogs [3–5], marine opisthobranchs [6], ladybird beetles [7,8] and paper wasps *Polistes dominula* [9]. The identification of these relationships has renewed interest in the ideas that warning signals may inform predators about levels of prey defence and that such signals are honest indicators of defensive capability [10]. Several theoretical studies have, however, predicted the opposite: that well-defended prey should reduce investment in signals, because signalling carries an additional conspicuousness cost, and such prey stand a good chance of surviving any attack unharmed [1–13]. Negative signal–defence correlations have been reported across some dendrobatid poison frogs [14] including *Dendrobates granuliferus* [15]. Using a novel theoretical model, Blount *et al.* [16] was able to explain both the positive and negative correlations between warning colours and chemical defences if these two traits are linked through the competitive use of a shared resource. One possible shared resource is energy, which can be limiting for the sequestration or biosynthesis of toxins [17]. Alternatively, sequestration of toxins could impose metabolic costs through oxidative lipid damage and reduced antioxidant defences—an idea that has received limited empirical attention in regard to warning signals.

**Figure 1.**
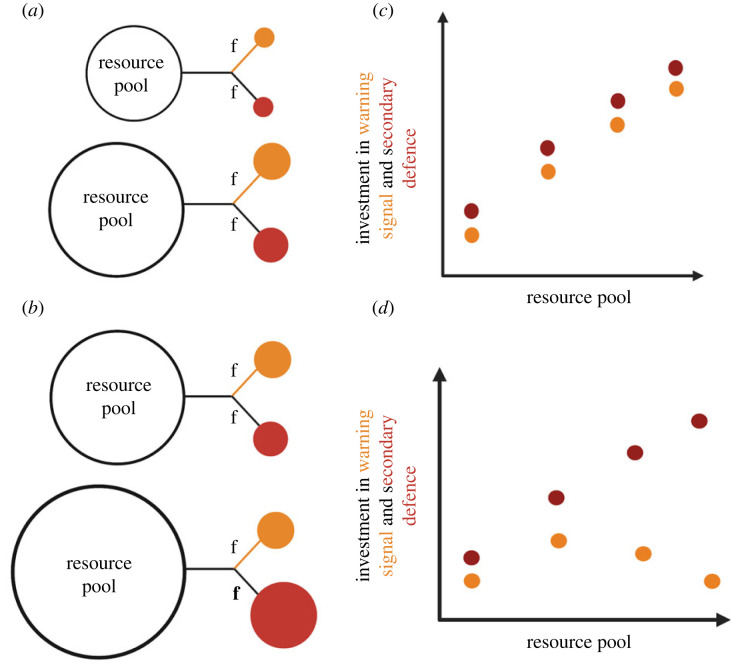
The resource allocation trade-off model by Blount *et al.* [16] predicts that individual prey acquire antioxidant resources from their environment (represented by the circle resource pool), which they divide via genetically or physiologically controlled flow rates (f) between sequestering defensive toxins (red circle) and in using antioxidants to produce pigments used for warning signals (orange circle). Each individual or population has access to a given level of resources (represented by the size of the circle) that they allocate to each defence type. In scenario (*a*), prey allocate resources equally to colour and toxicity. Those with more resources are able to allocate more resources than prey with a smaller resource pool, resulting in (*c*) a positive correlation between colour and toxicity, with only the most resource rich prey having the most colourful and toxic defences. In scenario (*b*), resource availability is very high for some prey (represented by the largest circle resource pool) and those prey with excess resources invest more in toxins than in warning signals (represented by the larger f and red circle), resulting in (*d*) a negative correlation between colour and toxicity. Blount *et al.* [16] suggest that this is because highly toxic prey can protect themselves sufficiently even with low levels of warning colours.

Many plant allelochemicals are powerful pro-oxidants, which, when ingested, can cause oxidative lipid damage [18]. In their resource competition framework, Blount *et al.* [16] envisaged that pigments used in prey warning signals (e.g. carotenoids, flavonoids, melanins, and pteridines) might play a dual role in imparting colour and acting as antioxidants that prevent self-damage when storing toxins. In their model, if antioxidants are required to enable individuals to withstand high levels of toxicity, signal reliability can be explained if the brightest and most toxic species gain access to more of the limiting resource than those that are less bright and less toxic (figure 1*a,c*). The prediction of signal honesty breaks down in the model when prey have very abundant resources, in which case it pays to divert these resources increasingly into toxins rather than warning signals, because less conspicuous but highly toxic prey encounter predators less often and have higher chances of surviving attacks (figure 1*b,d* [16]). Directly controlling antioxidant availability is challenging experimentally; consequently it is unclear whether colour–toxin relationships are influenced by the resource state of prey [14,15], and this has not yet been examined experimentally [11,12,16,17,19].

The monarch butterfly (*Danaus plexippus*) selectively metabolizes toxic cardenolides present in its host plants and stores them in its body [20,21]. We manipulated the availability of diet-derived toxins available to monarch caterpillars to test whether toxin sequestration and the production and maintenance of adult warning signals are linked via oxidative state. The various species of the monarch's larval milkweed host plants (Apocynaceae) are known to vary inter- and intraspecifically in cardenolide concentration and cardenolide polarity [21,22]. We test whether (i) the quantity of sequestered secondary defence by caterpillars is associated with levels of oxidative lipid damage and antioxidant defences in adults, and (ii) whether oxidative lipid damage incurred during sequestration affects the capacity of adult butterflies to produce aposematic warning signals.

Because there is substantial genetic variation in sequestration ability, and because evolutionary history and contemporary species interactions may influence patterns of cardenolide sequestration [23], we used monarchs from a single population. We reared monarchs on four milkweed species (table 1) that show within- and between-species, and among-population variation in cardenolide concentration, diversity and polarity [22,32–34]. Based on the published records of biological activity and chromatic quantification (e.g. HPLC and thin layer chromatography) of cardenolides of milkweeds in different populations and latitudes [35,36], our four plant species were used to represent a gradient of low to high cardenolide defences, with the aim of creating different costs of sequestration. We predicted that concentrations of cardenolides in the body and wings of newly eclosed adults would differ according to the host plant on which they were reared (see Material and methods): *Asclepias curassavica* (*Ac*) ≥ *A. syriaca* (*As*) > *A. incarnata* (*Ai*) ≥ *A. tuberosa* (*At*).

**Table 1.** The four species of *Asclepias* used in the rearing experiments with descriptions of their use by monarchs and phytochemistry.

*Asclepias* species	description and cardenolide content
*Asclepias curassavica*	a critical host plant worldwide and contains a diversity of cardenolides, including apolar voruscharin which is detrimental to monarch performance [21,24–27]
*A. syriaca* (common milkweed)	principal native host plant of the monarch east of the Rocky Mountains. Characterized by more polar cardenolides though also contains the apolar labriformin [28,29]
*A. incarnata* (swamp milkweed)	preferred host plant in the Midwest USA and has low concentrations of cardenolides [30]
*A. tuberosa* (butterfly milkweed)	an occasional native host plant in the wild with low concentrations of cardenolides [21,31]

Because sequestration, modification and storage of allelochemicals are believed to be oxidatively stressful for many chemically defended organisms [18], we predicted that body levels of oxidative lipid peroxidation would differ among individuals according to host plant, as follows: *Ac* ≥ *As* > *Ai* ≥ *At*. In turn, we predicted that survival to eclosion would differ according to host plant: *At* ≥ *Ai* > *As* ≥ *Ac*, and that the redness, luminance and conspicuousness of the warning signals would differ: *Ac* ≥ *As* > *Ai* ≥ *At*. In addition to testing for treatment effects, we examined associations between individual levels of cardenolides, oxidative state, and warning signal redness, luminance and conspicuousness. We predicted a positive association between levels of cardenolides and lipid peroxidation, a negative association of cardenolides with antioxidant defences, and that highly toxic prey would be the most conspicuous [16].

## 2. Material and methods

### (a) Milkweed plants

Milkweeds (*A. incarnata, A. tuberosa, A. curassavica* and *A. syriaca*) were locally sourced in Amherst, Virginia, USA. We did not measure cardenolide content in the leaves of the plants used in the experiment, but used published records about the spatial repeatability of cardenolide defences that take into account latitudinal clines [35–37]. From these records, we predicted that for milkweeds grown in Mid-Atlantic and Southeastern regions of the USA, total cardenolide content would vary in the order *Ac ≥ As > Ai ≥ At*. The plants were grown without the use of pesticides [38]. All plants were healthy with undamaged leaves at the start of the experiment.

### (b) Milkweed feeding assay and preparation of samples

Eggs were obtained from wild caught monarch females in Virginia (USA) that were kept in captivity until they laid eggs [39]. Using a split family design, larvae were reared by LPB on single plants under the same conditions. All larvae had abundant food during the experiment. Larvae were monitored daily for pupation starting at day 12.

Upon eclosion, the date and the individual's sex were recorded. Adults were placed in glassine envelopes and frozen on dry ice until dead, then stored in a freezer at −80°C before being shipped to the UK on dry ice. Adults were stored at −80°C in J.D.B.'s laboratory until they were analysed.

For analysis, the gut was removed, and the head, thorax and abdomen (hereafter, ‘body’) were bisected longitudinally. One half of the body was returned to storage at −80°C for later analysis of cardenolides. The other half of the body was homogenized in ice-cold phosphate-buffered saline (PBS; 5% w/v), for analyses of oxidative stress. The wings were removed using dissecting scissors and shipped on dry ice to H.M.R.'s laboratory for analyses of coloration (see below). Wings were then returned on dry ice to J.D.B.'s laboratory for cardenolide analysis.

### (c) Determination of oxidative stress and cardenolide concentration

Determining oxidative state requires a range of assays including antioxidant defences and oxidative damage. We assayed three such components: total superoxide dismutase (SOD), which are metalloenzymes that catalyse the dismutation of superoxide into oxygen and hydrogen peroxide and form a crucial part of intracellular antioxidant defences; total antioxidant capacity (TAC), which measures the activity of low molecular weight chain-breaking antioxidants including ascorbate, a-tocopherol, carotenoids and flavonoids, and therefore gives an overview of the antioxidant status of individuals (including diet-derived antioxidants); and malondialdehyde (MDA), which is formed by the β-scission of peroxidized polyunsaturated fatty acids, and therefore is a definitive marker of oxidative damage.

#### (i) Total superoxide dismutase

SOD was assayed by measuring the dismutation of superoxide radicals generated by xanthine oxidase and hypoxanthine. We followed the instructions of the kit (no. 706002; Cayman Chemical, Michigan, USA), except that tissue homogenates were further diluted with the supplied sample buffer (1 : 100 v/v) to ensure that SOD activity fell within the range of the standard curve. Samples were assayed in duplicate and are reported as units of SOD activity (U) per mg tissue.

#### (ii) Total antioxidant capacity

TAC was assayed by measuring the capacity of tissue homogenate to inhibit the oxidation of 2,2'-azino-di-[3-ethylbenzthiazoline sulfonate] (no. 709001; Cayman Chemical). The homogenate was further diluted with sample buffer (1 : 5 v/v) to bring the absorbance values within the range of the standard curve. Samples were assayed in duplicate, as per the kit instructions. Data are reported as nanomoles of TAC activity (Trolox equivalents) per gram tissue.

#### (iii) Malondialdehyde

MDA was measured using HPLC with fluorescence detection as described previously for tissue homogenates (e.g. [40]). All chemicals were HPLC grade, and chemical solutions were prepared using ultrapure water (Milli-Q Synthesis; Millipore, Watford, UK). Samples were derived in 2 ml capacity polypropylene screw-top microcentrifuge tubes. For tissue homogenates and for the standard (1,1,3,3-tetraethoxypropane, TEP; see below) a 20 µl aliquot was added to 20 µl butylated hydroxytoluene solution (BHT; 0.05% w/v in 95% ethanol), 160 µl phosphoric acid solution (H_3_PO_4_; 0.44 *M*), and 40 µl thiobarbituric acid solution (TBA; 42 mM). Samples were capped, vortex mixed for 5 s, then heated at 100°C for 1 h in a dry bath incubator to allow formation of MDA-TBA adducts. Samples were then cooled on ice for 5 min, before 80 µl *n*-butanol was added and tubes were vortex mixed for 20 s. Tubes were centrifuged at 12 000*g* and 4°C for 3 min, before the upper (*n*-butanol) phase was collected and transferred to an HPLC vial for analysis. Samples (10 µl) were injected into a Dionex HPLC system (Dionex Corporation, California, USA) fitted with a 5 µm ODS guard column and a Hewlett-Packard Hypersil 5µ ODS 100 × 4.6 mm column maintained at 37°C. The mobile phase was methanol buffer (40 : 60, v/v), the buffer being a 50 mM anhydrous solution of potassium monobasic phosphate at pH 6.8 (adjusted using 5 M potassium hydroxide solution), running isocratically over 3.5 min at a flow rate of 1 ml min^−1^. Data were collected using a fluorescence detector (RF2000; Dionex) set at 515 nm (excitation) and 553 nm (emission). For calibration, a standard curve was prepared using a TEP stock solution (5 mM in 40% ethanol) serially diluted using 40% ethanol. Data are presented as nanomoles MDA per gram tissue.

#### (iv) Cardenolide analysis

For cardenolide analysis, samples (wings and the remaining half of the body) were dried in an oven at 60°C for 16 h. The dried material was ground to a fine powder using a pestle and mortar, and weighed to the nearest 0.0001 g using a GR-202 electronic balance (A&D Instruments Ltd, Abington, UK). The samples were placed in screw-top polypropylene tubes for de-fatting and cardenolide extraction. To remove fats, 2 ml hexane was added and samples were incubated at 35°C for 30 min, before being centrifuged at 10 000*g* and 4°C for 5 min; the supernatant was collected and discarded. Ethanol (1.9 ml) containing 20 µg digitoxin (no. D5878; Sigma-Aldrich, Dorset, UK) as an internal standard were added to the pellet and it was briefly vortexed to extract cardenolides. Samples were placed in an XUB5 ultrasonic bath (Grant Instruments, Shepreth, UK) at 60°C for 60 min, followed by further centrifugation (9600*g* and 4°C for 5 min). The supernatant was removed and evaporated to dryness using a Savant ISS 110 SpeedVac Concentrator at room temperature (Thermo Scientific, Altrincham, UK). Samples were then dissolved in 0.5 ml methanol by vortexing, and 50 µl of the solution was injected into a Dionex HPLC system fitted with a Waters Spherisorb 5 µm ODS2 column (150 × 4.6 mm) maintained at 20°C. A multistep gradient of acetonitrile (ACN) and ultrapure water was used as the mobile phase, as follows: initial concentration 20% ACN held for 5 min, ramp to 70% ACN at 20 min and held until 25 min, ramp to 95% ACN at 30 min and held until 35 min, returning to 20% ACN at 40 min, with an end time of 50 min. The flow rate was 0.7 ml min^−1^. Peaks were collected using a PDA-100 photodiode array detector (Dionex) at an absorbance value of 218 nm. Spectral data between 200–400 nm were collected, and cardenolides were identified as peaks with a symmetrical absorbance band with a maximum absorbance between 217 and 222 nm [41,42]. Data are presented as micrograms of total cardenolides per 0.1 g tissue.

### (d) Measurements and visual modelling of coloration

The reflectance of the forewings of each butterfly, and of four leaves from *Asclepias syriaca* (as a representative background that the butterflies can rest on. Obtained from A. Agrawal), were measured using an Ocean Optics USB2000 spectrophotometer, with specimens illuminated at 45° to normal by a DH1000 balanced halogen deuterium light source. The measuring spot diameter was 3 mm, with spectra recorded at 0.34 nm intervals from 300 to 700 nm and measured relative to a WS-1 reflectance standard. For each wing, three to four non-overlapping measurements were taken per orange segment of the wing (electronic supplementary material, figure S1; total number of measurement spots per forewing = 13–14). For the leaves, we took 11 non-overlapping measurements. Spectrophotometry data were recorded using Ocean Optics OOBase32.

To determine differences in chroma between treatments, we modelled the predicted photon catches of a generalized passerine bird (passerines being the main avian predators of monarchs [43]) for each spectrum following the Vorobyev–Osorio model [44], using the R package PAVO [45]. We used the cone types of a blue tit, *Cyanistes caeruleus* [46]: longwave, LWS, *λ*max 563 nm; mediumwave, MWS, *λ*max 503 nm; shortwave, SWS, *λ*max 448 nm; ultraviolet, UVS, *λ*max 371 nm; and double dorsal, DD *λ*max 563 nm. We calculated redness as the ratio of long wave to medium wave cone responses. We also calculated a measure of chromatic conspicuousness as *Δ*S between the mean colour of the forewings of the butterflies and mean colour of the *A. syriaca* leaves using the Vorobyev–Osorio colour discrimination model, which is based on evidence that colour discrimination is determined by noise arising in the photoreceptors and is independent of light intensity. We used a Weber fraction value of 0.05 for the most abundant cone type [47]. We calculated luminance as the response of the dorsal double cone [48].

### (e) Data analyses

Left- and right-wing cardenolide concentration were significantly positively correlated (*r* = 0.96, *t* = 20.75, d.f. = 38, *p* < 0.0001). There was no significant correlation between asymmetry in wing cardenolide concentrations and the mean cardenolide concentration of the wings (*r* = 0.10, *t* = 0.64, d.f. = 38, *p* = 0.52), and so for each individual we calculated a mean wing cardenolide concentration. Left- and right-wing redness were significantly positive correlated (*r* = 0.85, *t* = 10.27, d.f. = 38, *p* < 0.0001), as were left- and right-wing conspicuousness (*r* = 0.86, *t* = 0.33, d.f. = 38, *p* < 0.0001), and left- and right-wing luminance (*r* = 0.47, *t* = 3.32, d.f. = 38, *p* = 0.002). For each individual we calculated a mean forewing redness, conspicuousness and luminance.

We log transformed MDA and wing and body cardenolide concentration to normalize the distribution of the residuals. We analysed the effect of host plant on total cardenolide concentration, body cardenolide concentration and wing cardenolide concentration using a GLM with a Gaussian distribution and identity link function. We set *A. tuberosa* as the species against which we compared the other plants, because of its low cardenolide content. Eclosion success or failure was analysed with a binomial GLM. To analyse how cardenolides and oxidative stress affected colour metrics in the butterflies we constructed a full model that included all pairwise interactions and a three-way interaction between MDA × total cardenolide concentration × sex and compared this to a simple model that included only the fixed effects of MDA, total cardenolide concentration and sex. We compared the reduced model to the full model by using information criteria (AIC; see electronic supplementary material, tables S1–S13). We visualized the interaction between two continuous variables using the rsm package in R [49]. All data were analysed using R version 4.0.4.

## 3. Results

### (a) Cardenolide concentrations

**Figure 2.**
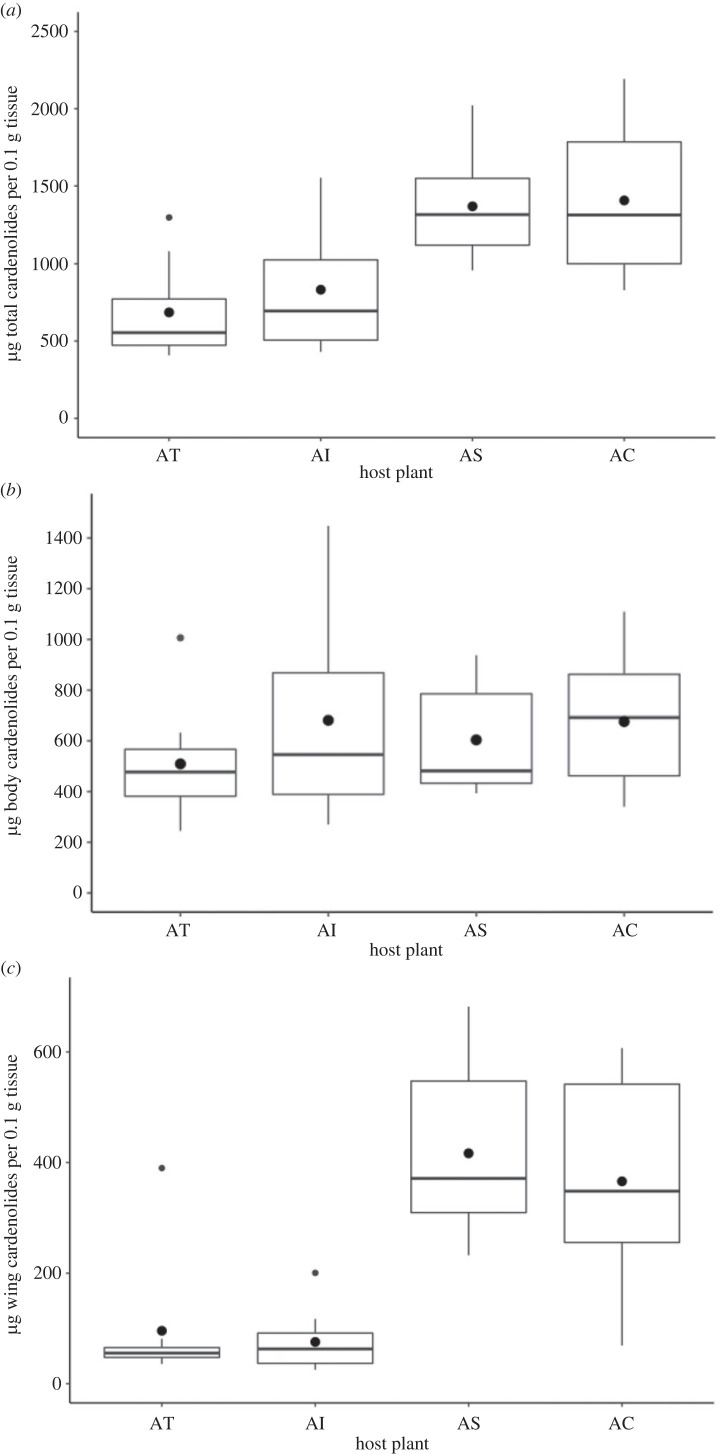
Cardenolide sequestration by monarchs feeding on *Asclepias tuberosa* (AT), *A. incarnata* (AI), *A. syriaca* (AS), and *A. curassavica* (AC); (*a*) total cardenolide (micrograms of total cardenolides per 0.1 g tissue) measured in the wings and bodies, (*b*) body cardenolide content, (*c*) wing cardenolide content. Boxplots show the median, interquartile range and the whiskers represent the largest and smallest value within 1.5 times the 25th and 75th percentiles. Outliers are represented by small black dots. Mean cardenolide concentrations are represented by large black dots.

In support of our first hypothesis, concentrations of cardenolides in the body and wings of newly eclosed adults differed according to the host-plant species on which they were reared (figure 2*a*; *F*_4,36_ = 7.42, *p* = 0.0002). Monarchs reared on *Ac* and *As* sequestered significantly more cardenolides in total than those reared on *At* (*Ac* versus *At:* estimate = 822.9 ± 184.3, *t* = 4.47, *p* < 0.0001; *As* versus *At*: estimate = 726.6 ± 187.1, *t* = 3.88, *p* = 0.0004). Those reared on *Ai* did not sequester significantly different total amounts of cardenolides than those on *At* (estimate = 213.9 ± 189.2, *t* = 1.13, *p* = 0.27). Males tended to sequester more cardenolides in total than females, but this difference was not significant at the alpha 0.05 level (estimate = 243.5 ± 133.4, *t* = 1.825, *p* = 0.076).

Body cardenolide concentration tended to differ between host plants, but the difference was not significant at the alpha 0.05 level (figure 2*b*; *F*_4,37_ = 2.471, *p* = 0.061). Wing cardenolide concentration significantly varied between host plants (figure 1*c*; *F*_4,36_ = 19.25, *p* < 0.001; figure 2*c*). Wing cardenolides were significantly higher in *A*c and *As* compared to *At* (*Ac* versus *At*: estimate = 1.59 ± 0.29, *t* = 5.48, *p* < 0.0001; *As* versus *At*: estimate = 1.78 ± 0.30, *t* = 6.05, *p* ≤ 0.001), but did not differ significantly between *At* and *Ai* (estimate = −0.05 ± 0.29, *t* = −0.18, *p* = 0.38).

### (b) Survival to pupation and eclosion

We predicted that sequestration of cardenolides would impose a cost that would be associated with reduced survivorship to eclosion. Successful eclosion was significantly higher in monarchs reared on *At* (89%) compared with those reared on *As* (20%; estimate = −2.06 ± 0.751, *t* = 2.75, *p* = 0.009), while there was no significant difference between *Ai* (70%) and *At* (89%, estimate = −0.696 ± 0.728, *t* = −0.956, *p* = 0.34) or *At* (89%) and *Ac* (62%; estimate = −0.927 ± 0.690, *t* = −1.344, *p* = 0.19). Survival to pupation did not differ significantly according to host-plant species (0% *At*, 23% *Ai*, 33% *As*, 27% *Ac*; Fisher's exact test *p* = 0.28).

### (c) Cardenolide and oxidative stress

#### (i) Malondialdehyde

**Figure 3.**
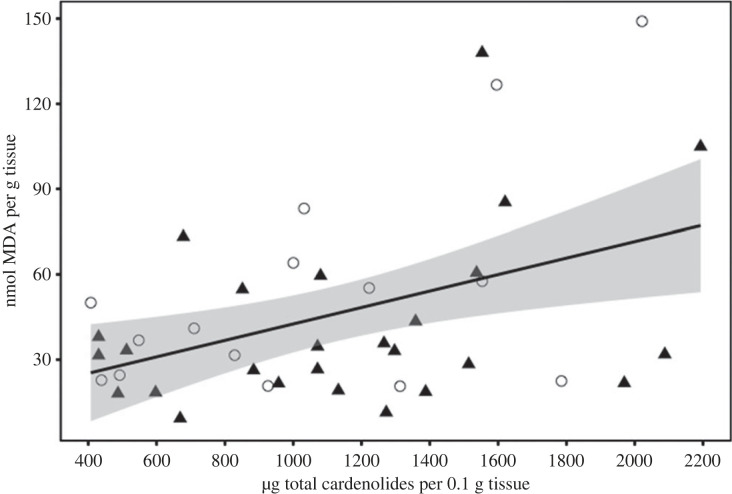
Total cardenolide concentration for males (filled triangle) and females (open circle) and malondialdehyde (MDA) concentration. Line is the smoothed conditional mean with 95% confidence intervals.

Contrary to our prediction that oxidative lipid damage would differ by host plant, we found no significant effect of host plant or sex on MDA (main effect *F*_4,37_ = 0.77, *p* = 0.55). In line with our prediction of a positive association between concentration of sequestered cardenolides and markers of oxidative damage, across all treatment groups individuals that sequestered higher total concentrations of cardenolides had higher levels of MDA (figure 3; main effect: *F*_2,38_ = 4.08, *p* = 0.025; cardenolide: estimate = 0.0005 ± 0.0002, *t* = 2.51, *p* = 0.016). There was no significant difference between the sexes (estimate = −0.3097 ± 0.198, *t* = −1.57, *p* = 0.13).

#### (ii) Superoxide dismutase activity

There was no significant effect of host plant or sex on SOD activity (main effect *F*_4,37_ = 0.28, *p* = 0.89). We found no significant effect of total cardenolide concentration or sex on SOD activity (main effect: *F*_2,38_ = 0.52, *p* = 0.60).

#### (iii) Total antioxidant capacity

There was no significant effect of host plant or sex on TAC (*F*_4,37_ = 0.42, *p* = 0.79) or total cardenolide concentration (*F*_2,38_ = 0.19, *p* = 0.83).

#### (iv) Sequestration, oxidative stress and warning signals

**Figure 4.**
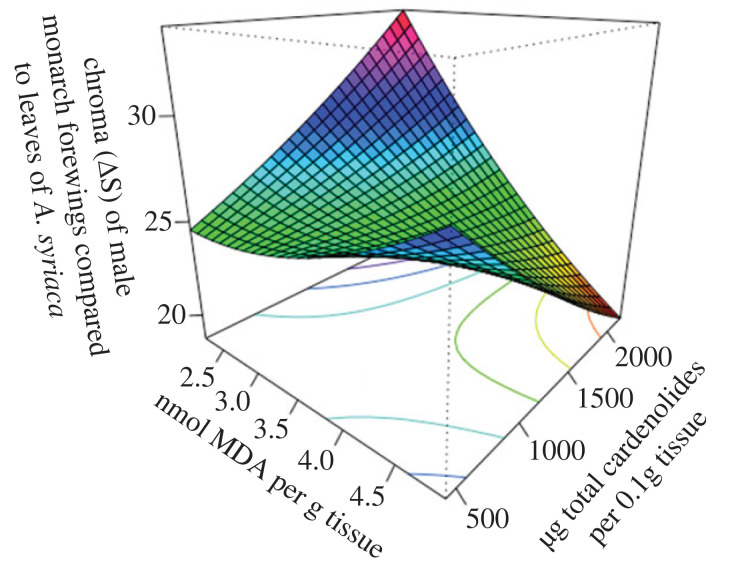
Three-dimensional surface (perspective) plot for the response surface predicting the relationship between the conspicuousness of the male monarch's forewing, oxidative damage measured as malondialdehyde (MDA) and total cardenolide concentration.

Because MDA was the only marker associated with individual levels of sequestration, we analysed its association with warning signals, but did not conduct analyses on SOD activity or TAC effects on warning signals*.* Monarchs varied significantly in conspicuousness (chroma (ΔS): *F*_3,35_ = 17.85 *p* < 0.0001). Males were significantly more conspicuous in chroma (*Δ*S) than females (estimate = 5.30 ± 0.82, *t* = 6.49, *p* < 0.001), and both sexes decreased in conspicuousness with increasing MDA, though this difference was not significant at the alpha 0.05 level (estimate = −1.26 ± 0.65, *t* = −1.95, *p* = 0.06). There was no significant effect of cardenolide concentration on wing conspicuousness (estimate = −0.00002 ± 0.0008, *t* = −0.03, *p* = 0.98). Because of the significant sexual dimorphism in colour and sequestration, we separated the analysis by sex and found a significant two-way interaction between MDA and total cardenolide concentration on the conspicuousness (chroma *Δ*S) of male forewings (main effect: *F*_3,21_ = 8.54, *p* = 0.0007; MDA × cardenolide: estimate = −0.003 ± 0.001, *t* = −3.24, *p* = 0.004) that is represented by a curved surface (figure 4). Males with the highest levels of sequestered cardenolides had the most conspicuous warning signals when oxidative damage was lowest, and reduced their warning signal conspicuousness as oxidative damage increased, while those that sequestered the lowest levels of cardenolides showed no change in warning signal conspicuousness across all levels of oxidative damage. The conspicuousness of males' warning signals decreased with increasing sequestration in males with high oxidative damage, whereas conspicuousness increased in males with low oxidative damage with increasing sequestration. There was no significant effect of oxidative damage or concentration of sequestered cardenolides on female forewing conspicuousness (*F*_3,10_ = 0.06, *p* = 0.98).

Males also had significantly redder forewings than females (main effect: *F*_3,35_ = 5.81, *p* = 0.002; sex estimate = 0.24 ± 0.06, *t* = 3.82, *p* = 0.0005), but there was no significant effect of MDA or cardenolide concentration on male and female redness (MDA: estimate = −0.04 ± 0.049, *t* = −0.86, *p* = 0.40; cardenolide concentration: estimate = 0.00001 ± 0.00006, *t* = 0.16, *p* = 0.87). We found a significant difference in brightness (*F*_3,35_ = 10.19, *p* = 0.00006). Males were significantly brighter than females (estimate = 0.03 ± 0.007, *t* = 4.37, *p* = 0.0001) and both sexes decreased in brightness with increasing MDA (estimate = −0.01 ± 0.005, *t* = −2.40, *p* = 0.02). There was no significant effect of cardenolide concentration on wing brightness (estimate = 0.000003 ± 0.000007, *t* = 0.37, *p* = 0.70).

## 4. Discussion

We reared monarch caterpillars on whole plants from single plant populations of four milkweed species with varying phytochemistry (*Asclepias tuberosa*, *A. incarnata*, *A. syriaca* and *A.curassavica*; [50]). Monarchs that sequestered higher concentrations of cardenolides experienced higher levels of oxidative damage than those that sequestered lower concentrations (measured as the concentration of malondialdehyde; MDA). Although there is some evidence that cardenolides can be a burden for monarch caterpillars [22,51], our results are among the first to show a potential physiological mechanism of oxidative damage as a cost of sequestration for monarchs (see also [21,37]). We found a trade-off between sequestration and conspicuousness in male monarchs mediated by oxidative damage. Males with high oxidative damage had decreasing conspicuousness with increasing sequestration, whereas males with low oxidative damage showed increased conspicuousness with increasing sequestration. This is the first evidence to support Blount *et al.*'s [16] resource allocation trade-off model—prey with lower oxidative damage are able to allocate more resources to colour and toxicity than prey with higher oxidative damage—and shows that an ability to deal with oxidative damage can result in colour–toxin correlations in monarch butterflies.

Some authors [52] have rejected the idea that there can be a mechanistic link between warning signals and chemical defences that involve intrinsic costs of production or maintenance of defences because there was no clear mediator. However, condition-dependent expression of colourful signals can arise when the underlying physiological pathways that produce coloration are dependent on the same core cellular processes [16,53]. We found stable levels of antioxidant defences (superoxidase dismutase and TAC) but increased oxidative damage was associated with reduced warning signal conspicuousness, which suggests that the monarchs that are better able to control pro-oxidant levels can sequester more cardenolides and still invest in warning signals [54]. Similar patterns are observed in *Aristolochia*-feeding pipevine swallowtail butterflies (*Battus philenor*) that store nitrophenanthrenes and have significantly higher tissue levels of carotenoids than related species which mimic them in coloration but do not store toxins [55]. Similarly, the rank order of body concentrations of carotenoids in three species of lepidoptera matches the rank order of dietary exposure to the pro-oxidant toxin furanocoumarin (8-MOP) (*Papilio polyxenes* > *Spodoptera eridania* > *Trichoplusia ni*; [18]). Antioxidants are involved in detoxification processes [56] as well as pigment synthesis pathways directly [57], or as cofactors of enzymes [58]. Our results allow us to speculate that antioxidant availability has a role in the biochemistry underlying the variations in warning coloration and toxicity in aposematic animals. Warning colours are, however, usually regulated by more than one mechanism, and we suggest that this area of research warrants further biochemical study.

Oxidative state likely depends on the combination of genetic, environmental and gene by environment (G × E) interactions that determine an individual's condition. Monarchs show patterns of local adaptation to their host plants (based on larval growth rate [37]), and also show G × E interactions in sequestration ability [23] which may reflect either a lack of evolutionary history with different species of *Asclepias*, or a physiological trade-off in sequestration ability. Our results suggest that populations of monarchs that are sympatric with high cardenolide milkweeds could be less subject to oxidative damage, either because they have evolved higher antioxidant efficiency, or because they are able to accumulate more antioxidants to provide enhanced antioxidant protection. Further research on this topic could have interesting implications for our understanding of host shifts, range expansions and automimicry (the occurrence of palatable ‘cheaters’ in a chemically defended population). Whether automimicry in monarchs arises directly from a deficiency in an individual's capacity to cope with reactive oxygen species warrants further study [59].

Our results depend, to some extent, on the measure of signal conspicuousness measured against the leaves of one of the four host plants (*A. syriaca*), and the measurement of total cardenolide content by high-performance liquid chromatography. The differences in conspicuousness of the forewings to the host-plant leaves range from (ΔS) 14 to 29. These ‘suprathreshold’ colour differences are often assumed to scale linearly with colour distance, but this is not the case in some visual systems [60]. Experiments testing predator responses to the colour differences, and the consequences for prey survival, will be important for determining whether the levels of measured conspicuousness result in differential survival. Future research could also calculate conspicuousness against a range of host-plant species and plant parts, as well as considering predator visual acuity and the effects of increasing viewing distance on warning signal conspicuousness. The total cardenolide content of monarchs in our study ranged from 400 to 2100 µg per 0.1 g of tissue. Predators would likely respond differently to these levels, because Brower [32] determined that similar variation to that which we report here represents a spectrum of toxicity to predatory blue jays (measured as the number of blue jays that would be made unwell by consuming the butterflies). A more mechanistic quantification of toxicity to predators could be employed, i.e. using *in vitro* tests of the cellular target of cardenolides, the sodium–potassium pumps (Na^+^/K^+^ -ATPase), which could determine if the cardenolide concentrations of the monarchs represent physiologically different levels of toxicity. Our future work will address whether predators are a source of selection for the cardenolides sequestered by milkweed herbivores. Together, our results suggest that predators have the potential to distinguish between monarchs that vary in toxins and warning signals. This could result in selection against moderately defended and moderately conspicuous individuals, because predators may visually reject highly conspicuous prey, and taste reject highly defended but less conspicuous prey. Such differential selection could maintain the signal variability we observe in male monarchs and in other aposematic prey.

The resource competition model does not investigate or predict sex differences in resource limitation. However, Blount *et al.* [61] found that ladybird females (the larger sex) are more susceptible to resource limitation than males, and hence more likely to signal honestly. In this study we found costs of signalling in male monarchs. Males are slightly larger than females and are the more active sex. Males also sequestered more cardenolides than females. This behavioural difference between the sexes may increase males' probability of detection and risk of predation, and also increase their sensitivity to oxidative damage.

Although we did not measure cardenolide content in milkweed plants, our results relating to the sequestration patterns of monarchs supports previous research showing that the concentration of sequestered cardenolides varies depending on host-plant chemistry [62,63], and follows a standard approach to using the natural between species and population variability in plant toxicity to test for costs of sequestration [21,35,50]. However, it is methodologically difficult to separate the costs of sequestration from the potential effects of other plant defences and nutrient availability that vary between milkweed species [22,64]. Future work could focus on comparative phytochemical profiles to understand resource availability, and experiments that control nutritional content while manipulating toxin content [65].

In conclusion, our results support the proposal that oxidative state can be a key physiological mechanism that links warning colours to sequestration costs [16], and that the contribution of pigments to antioxidant activity for maintaining redox homeostasis is important [59]. Our results show that specialist herbivores must balance the benefits of plant secondary compounds for sequestration with the burden that these same compounds impose (though see [30,50]). Future research should examine the possibility of natural selection for oxidative capacity, and whether aposematism evolves in species that have high antioxidant capacity. These studies could provide new theories for understanding the evolution of aposematism when coloration and toxicity do not coevolve. Documenting the costs associated with using secondary defences in natural systems is important for our understanding of the ecology and evolution of aposematism.

## Data Availability

Data are available in the Open Research Data Repository of the Max Planck Society. PAVO colour analysis pipeline: https://doi.org/10.17617/3.GIXTT3 [66]. Data on oxidative state markers and coloration of monarch butterflies: https://doi.org/10.17617/3.YC1BR4 [67]. Data are provided in the electronic supplementary material [68].
